# Phloroglucinaldehyde Alleviates High-Fat-Diet-Induced MAFLD via Its Antioxidant and Anti-Inflammatory Properties

**DOI:** 10.3390/foods15030437

**Published:** 2026-01-25

**Authors:** Jijun Tan, Jianhua He, Hongfu Zhang, Shusong Wu

**Affiliations:** 1 Department of Animal Nutrition and Feed Science, School of Life Sciences and Environmental Resources, Yichun University, Yichun 336000, China; jijun995@jxycu.edu.cn; 2 Hunan Collaborative Innovation Center for Utilization of Botanical Functional Ingredients, Hunan Agricultural University, Changsha 410128, China; 3 State Key Laboratory of Animal Nutrition, Institute of Animal Sciences, Chinese Academy of Agricultural Sciences, Beijing 100193, China

**Keywords:** phloroglucinaldehyde, MAFLD, anthocyanins, polyphenol metabolites

## Abstract

Metabolic associated fatty liver disease (MAFLD), redefined from non-alcoholic fatty liver disease (NAFLD), is a global health concern driving the search for dietary interventions based on natural compounds. Phloroglucinaldehyde (PGA), a primary phenolic metabolite of the widely consumed anthocyanin cyanidin-3-glucoside (C3G) found in berries and other fruits, has emerged as a promising candidate due to its potential higher bioavailability than its parent compound. This study investigates the protective effects of PGA against high-fat diet (HFD)-induced MAFLD. Using both in vitro (LO2 cells) and in vivo (C57BL/6J mice) models, we found that PGA administration significantly attenuated body weight gain and hepatic steatosis, while reducing serum levels of TG, TC, liver transaminases (AST & ALT), and insulin resistance (*p* < 0.05). Further liver lipidomic profiling revealed that PGA supplementation specifically down-regulated 46 lipid species (*p* < 0.05), predominantly triglycerides characterized by long-chain and very-long-chain saturated fatty acids. Mechanistically, PGA enhanced the hepatic antioxidant capacity by increasing superoxide dismutase (SOD) activity (*p* < 0.05) and decreasing malondialdehyde (MDA) (*p* < 0.05) and exerted anti-inflammatory effects by reducing pro-inflammatory cytokines (IL-6, TNF, MCP-1) (*p* < 0.05) and endotoxin levels (*p* < 0.05). Correlation analyses further linked the down-regulated lipids to improvements in oxidative stress and inflammation. Our findings underscore that PGA, a key bioactive metabolite derived from dietary anthocyanins, alleviates MAFLD through its potent antioxidant and anti-inflammatory properties, highlighting its potential as a functional food ingredient or nutraceutical for metabolic health.

## 1. Introduction

The redefinition of non-alcoholic fatty liver disease (NAFLD) as metabolic associated fatty liver disease (MAFLD) marks a paradigm shift toward a positive diagnostic criterion based on underlying metabolic dysfunction. A meta-analysis involving 78 million population from 38 countries revealed its prevalence is approaching 30.2% [1]. MAFLD is strongly driven by modern “western-style” diets high in saturated fats and sugars [2]. The pathogenesis of MAFLD is well explained by the “multiple-hit” hypothesis [3], which describes its progressive nature. The “first hit” primarily involves insulin resistance [4,5], orchestrated by genetic, nutritional, and environmental factors. Insulin resistance-induced dysregulation of glycolipid metabolism contributes to accumulation of free fatty acids and de novo lipogenesis in liver [6]. However, this represents only the initial stage of MAFLD. The critical “subsequent hit” emerges from a lipotoxic environment within the liver. This toxicity directly triggers oxidative stress through dysfunction in mitochondrion [7], along with overproduction of reactive oxygen species [8]. Concurrently, these pathological changes activate a series of inflammatory actions, leading to the increase in pro-inflammatory cytokines and the establishment of chronic inflammation [9]. The cascade reactions between oxidative stress and inflammation form the core mechanism driving hepatocellular damage, apoptosis, and disease progression to more severe stages including steatohepatitis, and potentially hepatocellular carcinoma [10]. This pathological progression also explains why MAFLD frequently coexists with cardiovascular diseases [11], kidney disease [12], and Type 2 diabetes mellitus [13], all sharing common metabolic disturbances. Therefore, targeting oxidative stress and inflammatory responses represents a crucial therapeutic strategy for MAFLD intervention [14,15].

Dietary patterns rich in edible herbs [16] and fruits [17] are proposed as reliable and sustainable strategies for MAFLD prevention and management. The beneficial effects of these foods are largely attributed to their high content of bioactive compounds, particularly polyphenols, which have gathered significant attention for their antioxidant [18] and anti-inflammatory [19] properties. Based on the structure of phenolic hydroxyl groups, polyphenols act as hydrogen donors, effectively scavenging free radicals and chelating metal ions, thereby exerting potent antioxidant activity. Anthocyanins, a prominent class of natural polyphenols rich in dark-colored plants such as berries, grapes, and red cabbage, are widely consumed and studied for their health-promoting effects [20]. Cyanidin-3-glucoside (C3G) represents the most common dietary anthocyanins, known to mitigate lipid peroxidation and inflammation [21,22]. However, a significant challenge in utilizing parent polyphenols like C3G is their low systemic bioavailability [23]. While strategies like encapsulation aim to improve this [24], compelling evidence indicates that the health benefits are often mediated by their bioactive metabolites, which exhibit better absorption and stability [25,26,27,28]. Our previous research on protocatechuic acid, a metabolite of C3G, demonstrated its efficacy against MAFLD [29]. Following this rationale, this study focuses on PGA, another major and potential metabolite of C3G. We hypothesize that dietary supplementation with PGA can ameliorate HFD-induced MAFLD, and we aim to elucidate its function on lipid metabolism, oxidative stress, and inflammation, thereby evaluating its potential as a functional food-derived bioactive.

## 2. Materials and Methods

### 2.1. Diets and Reagents

Two diets designed in different energy levels were purchased from commercial company based on our previous study [29]. Supplementary Table S1 (or accessible via http://researchdiets.com/ (accessed on 14 July 2025)) details their complete nutritional profiles. Other reagents, including oleic acid, palmitic acid, and PGA were purchased from different companies, such as Shanghai Yuanye Bio-Technology Co., Ltd. (Shanghai, China), and Sigma-Aldrich (St. Louis, MO, USA).

### 2.2. Cell Culture, Viability Assay and Oil Red Stain

The LO2 was maintained in DMEM, with 10% FBS, 100 U/mL penicillin, and 100 μg/mL streptomycin. Cells were cultured at 37 °C in a humidified incubator with 5% CO_2_.

Cell viability was assessed by CCK-8 assay. Briefly, LO2 cells were cultured in 96-well plates at 1 × 10^4^ cells per well. After 24 h of adhesion, following treatments were used to replace the original medium: complete medium, medium containing 1 mM fatty acids (FAs, a 2:1 mixture of oleic acid and palmitic acid), or medium with PGA at concentrations ranging from 25 to 500 μM, for another 24 h. Prior to the CCK-8 assay, the original treatment medium was removed to avoid any interference from polyphenol redox activity. Subsequently, 100 μL complete medium that contains 10% CCK-8 was added to every well for all treatments. Finished incubation for 2 h at 37 °C, the solution in 96-well plates were detected at 450 nm.

To evaluate lipid accumulation, LO2 cells were cultured in 24-well plates at 1 × 10^5^ cells per well. Similar to the above, but with the following treatments: complete medium, 1 mM FAs, or 1 mM FAs co-incubated with 25, 100, or 500 μM PGA, for 24 h. Eventually, method of Oil Red O staining was used to detect the condition of lipids deposited in LO2.

### 2.3. Mouse Model and Experimental Design

The animal experiments were conducted following approval by the Institutional Animal Care and Use Committee of Hunan Agricultural University (Approval No. 2020034). Specific pathogen-free (SPF) male C57BL/6J mice, aged four weeks, were obtained from Hunan Slake Jingda Laboratory Animal Co., Ltd. (Changsha, China; The Animal License is No. SCXK-Xiang 2016-0002). During the experiment, mice were housed in isolated cages (Suzhou Fengshi Laboratory Animal Equipment Co., Ltd., Suzhou, China) containing sterilized poplar bedding. Environmental conditions for mice were standardized at 23.5 °C with a 12 h light/dark cycle through the settings of the equipment system, and all mice had unrestricted access to feed and water [29]. Bedding and water were refreshed every three days, and daily feed intake was recorded.

Twenty-four C57BL/6J mice (SPF class, male, 4 weeks of age) were accommodated for 1 week and then randomly divided into 4 groups (*n* = 6), which were fed a LFD (LFD group, fat recommendation refers to normal diet), a HFD (HFD group), a HFD containing 0.025% (*w*/*w*) of PGA (HFD + 0.025%PGA group, LPGA group), or a HFD containing 0.1% (*w*/*w*) of PGA (HFD + 0.1%PGA group, HPGA group, respectively, for 12 weeks. The dosages of PGA were based on our previous study [29].

### 2.4. Calculation of Energy Intake and Intraperitoneal Fat Ratio

We measured mouse body weight at 2-week intervals for 12 weeks (from week 0 to week 12) and used these data to construct a longitudinal growth curve. Daily average energy intake of mouse was calculated according to daily feed intake and energy unit value (Supplementary Table S1). Upon completion of the experimental period, all mice were fasted overnight, anesthetized using ether, and subsequently euthanized. The final weight was recorded, and the weight of intraperitoneal fat (excluding epididymal fat), was accurately weighed to calculate the percentage of intraperitoneal fat to body weight (BW).

### 2.5. Assessment of Structural Morphology in Adipose and Hepatic Tissues

Samples of intraperitoneal fat and liver tissue were fixed in 4% paraformaldehyde or a specialized fat fixative (G1119, Servicebio, Wuhan, China), respectively, for a minimum of 24 h prior to processing. Following processing in a Servicebio (Wuhan, China) G-L5/P5 automated tissue processor, tissue specimens were embedded in paraffin blocks. Sections of 4 μm thickness were then cut using a G-P1 microtome (Servicebio, Wuhan, China) and stained by the hematoxylin and eosin (H&E) method. For cryosectioning, liver tissues were cryoprotected in 15% sucrose solution before being sectioned into 8–10 μm thick slices using a cryostat (CM1950, Leica, Wetzlar, Germany) for Oil Red O staining. All stained sections were examined under a microscope (PANNORAMIC 250, 3DHistech, Budapest, Hungary), and adipocyte numbers were quantified in representative fields.

### 2.6. Assessment of Biochemical Parameters and Cytokine Levels in Serum

We collected blood samples and let them clot at room temperature for 30 min. Serum was subsequently harvested by centrifuging the samples at 1500× *g* for 10 min with an Eppendorf 5424 centrifuge (Hamburg, Germany). We measured the serum activities of triglycerides (TG), total cholesterol (TC), aspartate aminotransferase (AST), and alanine aminotransferase (ALT) utilizing a MINDRAY BS-200 automated chemistry analyzer (Mindray Bio-Medical Electronics Co., Ltd., Shenzhen, China), guided by the manufacturer’s protocols. The assessments of blood glucose and serum insulin were performed using commercially available kits. Specifically, a glucose assay kit (Shanghai Rongsheng Biotech, Shanghai, China) and a Mouse INS ELISA Kit (Elabscience, Wuhan, China) were utilized for the respective measurements. The homeostasis model assessment of insulin resistance (HOMA-IR) was calculated as follows [30].

HOMA-IR = fasting insulinemia (mU/L) × fasting glycemia (mg/dL)/405

The concentrations of inflammatory cytokines in serum, including interleukin-6 (IL-6), IL-10, monocyte chemoattractant protein-1 (MCP-1), interferon-γ (IFN-γ), tumor necrosis factor (TNF), and IL-12p70, were conducted in accordance with the manufacturer’s instructions, employing the BD CBA Mouse Inflammation Kit (552364, BD Biosciences, San Jose, CA, USA) on a BD Accuri C6 flow cytometer (BD Biosciences, San Jose, CA, USA).

### 2.7. Assessment of Oxidative Stress Parameters and Hepatic Endotoxin

Liver tissues were rapidly collected after sacrifice, snap-frozen in liquid nitrogen, and stored at −80 °C until analysis. A 10% (*w*/*v*) liver homogenate was prepared in normal saline. The activity of superoxide dismutase (SOD) and the level of malondialdehyde (MDA), a marker of lipid peroxidation, were measured using specific commercial assay kits (Nanjing Jiancheng Bioengineering Institute, Nanjing, China). Hepatic endotoxin content was determined with a Mouse ET ELISA Kit (Shanghai Enzyme-linked Biotechnology, Shanghai, China), adhering to the manufacturer’s guidelines.

### 2.8. Lipidomics Investigation

Comprehensive lipidomic characterization was conducted based on our previous study [29]. Briefly, lipids were extracted from liver tissue using a methyl tert-butyl ether/methanol system, followed by comprehensive lipidomic profiling using a liquid chromatography-tandem mass spectrometry (LC-ESI-MS/MS) system. Chromatographic separation was achieved on a Thermo C30 column with a tailored gradient elution, and mass spectrometric detection was performed in multiple reaction monitoring (MRM) mode with optimized parameters for each lipid species. Lipids with a fold_change ≥ 2 or ≤0.5 were selected, and those with a variable importance in projection (VIP) absolute value ≥ 1 were considered statistically significant (*p* < 0.05), thereby identifying differential lipid metabolites between the two groups.

### 2.9. Statistical Analysis

Data are presented as mean ± standard deviation (SD). Differences among groups were evaluated by one-way analysis of variance (ANOVA) followed by post hoc tests (Fisher’s LSD and Duncan’s Multiple Range Test) using SPSS software (Version 21.0, IBM Corp., New York, NY, USA). Relationships between oxidative stress markers, inflammatory factors, and hepatic lipids were examined using Pearson’s correlation analysis. A *p*-value of less than 0.05 was considered statistically significant.

## 3. Results

### 3.1. PGA Attenuates Lipid Accumulation in LO2 Hepatocytes (In Vitro)

**Figure 1:**
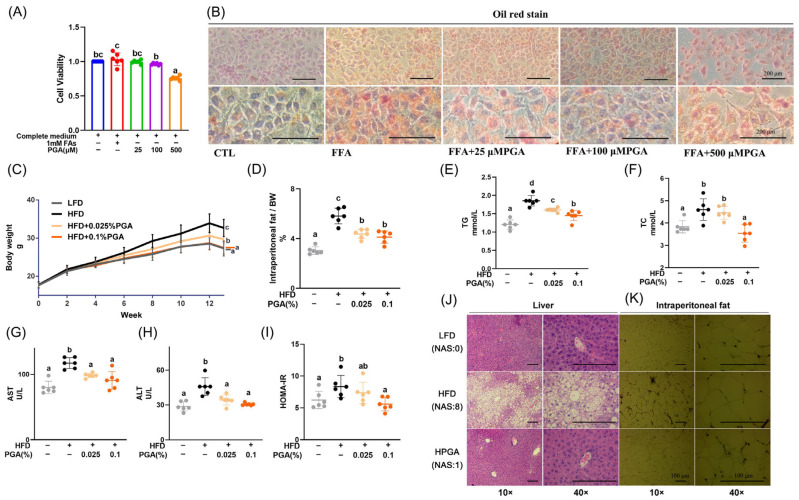
Effects of PGA supplementation on lipid deposition. (**A**,**B**) In vitro study: (**A**) Viability of LO2 cells treated with fatty acids (FAs; oleic acid–palmitic acid = 2:1) and PGA (25–500 μM), assessed by CCK-8 assay (*n* = 6). (**B**) Representative sections of LO2 cells by Oil Red staining after treatment with complete medium, complete medium & FAs, or complete medium & FAs & 25~500 μM PGA. (**C**–**K**) In vivo study: (**C**) Body weight growth curves, (**D**) intraperitoneal fat mass to body weight ratio, and (**E**–**I**) Serum levels of TG (**E**), TC (**F**), AST (**G**), ALT (**H**), and (**I**) HOMA-IR index for insulin resistance. (**J**,**K**) representative H&E-stained sections of liver (**J**) and intraperitoneal fat (**K**) (original magnification ×10 and ×40 objective, respectively) from C57BL/6J mice fed a HFD with or without PGA (*n* = 6). Bars labeled with different letters indicate significant differences (*p* < 0.05). Hepatic histopathological scores were evaluated based on NAFLD Activity Score (NAS). The score ranging from 0 to 8 is defined as the unweighted sum of the scores for steatosis (0–3), lobular inflammation (0–3) and ballooning (0–2).

We first investigated the direct effects of PGA on hepatocyte steatosis and viability using an in vitro model. Treatment with a mixture of fatty acids (FAs, 1 mM) did not affect cell viability compared to CTL group (Figure 1A, *p* > 0.05), and induced lipid accumulation in LO2 cells, as visualized by Oil Red O staining (Figure 1B). Co-treatment with PGA at 25 μM did not affect cell viability compared to either the CTL or FA-only groups (Figure 1A, *p* > 0.05), and alleviated FAs-induced lipid deposition in hepatocytes (Figure 1B). While 100 μM PGA resulted in a significant reduction in viability compared to the FA-only group (Figure 1A, *p* < 0.05), its absolute viability was not significantly different from the CTL baseline (Figure 1A, *p* > 0.05), and it effectively reduced lipid accumulation (Figure 1B). The higher concentration (500 μM PGA) exhibited significant cytotoxicity compared to all other groups (Figure 1A,B, *p* < 0.05). These in vitro findings demonstrated that PGA, at non-cytotoxic concentrations (25–100 μM), directly reduces lipid accumulation in hepatocytes.

### 3.2. PGA Supplementation Ameliorates HFD-Induced MAFLD Phenotypes in Mice (In Vivo)

Based on the effects observed in vitro, we next evaluated the efficacy of PGA in a diet-induced MAFLD mouse model. Analysis of daily energy intake revealed a specific pattern: no significant difference was found among the LFD, HFD, and HPGA groups (*p* > 0.05; Supplementary Figure S1A), indicating that the HFD-induced MAFLD was driven by diet composition rather than increased caloric consumption. In contrast, the LPGA group consumed significantly more energy than the HFD and HPGA groups (*p* < 0.05; Supplementary Figure S1A). As expected, consumption of a high-fat diet (HFD) for 12 weeks significantly increased final body weight (BW) (*p* < 0.05; Figure 1C) and the intraperitoneal fat rate, a key index of visceral adiposity (*p* < 0.05; Figure 1D). Histological analysis confirmed severe hepatic steatosis (Figure 1J) and enlarged intraperitoneal adipocytes (Figure 1K and Supplementary Figure S1B) in the HFD group.

Furthermore, HFD feeding induced systemic metabolic disturbances, marked by elevated serum levels of TG, TC (*p* < 0.05; Figure 1E,F), liver transaminases (AST and ALT, *p* < 0.05) (Figure 1G,H) and increased insulin resistance, as indicated by a higher HOMA-IR index (*p* < 0.05; Figure 1I), which was supported by altered serum glucose and insulin levels (Supplementary Figure S1C,D).

Notably, dietary supplementation with PGA, especially 0.1% (HPGA) doses, significantly reversed these adverse metabolic phenotypes. PGA treatment attenuated body weight gain, reduced intraperitoneal fat accumulation, alleviated hepatic steatosis, and improved insulin sensitivity and liver function markers (*p* < 0.05; Figure 1C–I). These results unequivocally demonstrate the potent efficacy of PGA in counteracting diet-induced obesity and MAFLD progression in vivo.

### 3.3. Dietary Supplement of PGA Down-Regulated Forty-Six Types of Differential Hepatic Lipid Metabolites

To elucidate the impact of PGA on lipid metabolism, we performed a comprehensive lipidomic analysis on liver tissue. Compared to the LFD group, the HFD regimen significantly altered the hepatic lipid profile, up-regulating 120 and down-regulating 41 lipid species (*p* < 0.05; Supplementary Figure S1E & Supplementary Table S2).

**Figure 2:**
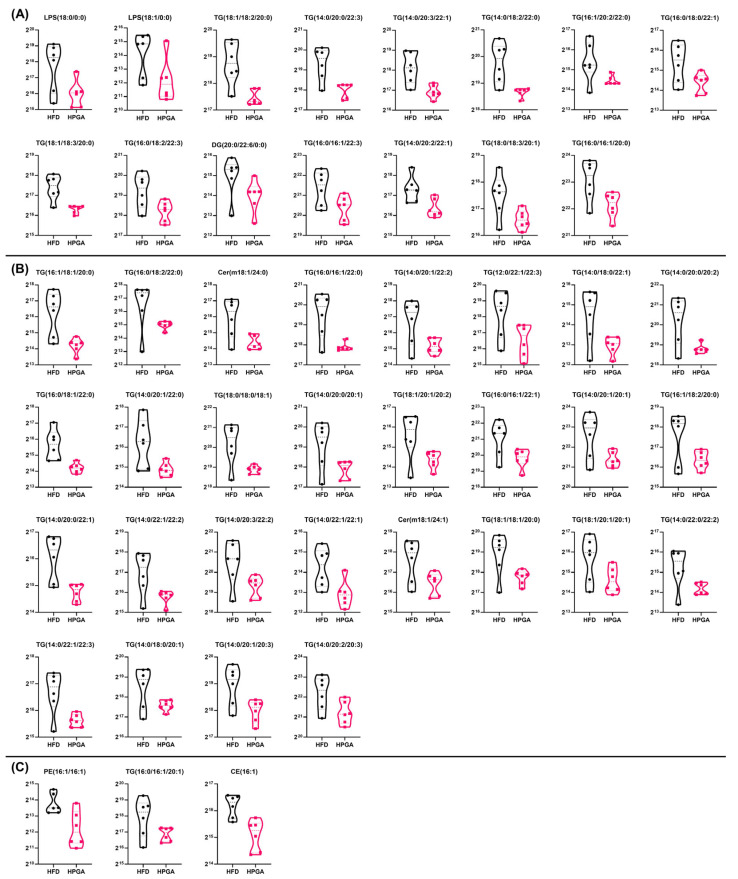
Forty-six types of significant lipid metabolites down-regulated by dietary supplementation of PGA (*p* < 0.05). Comprehensive lipidomic profiling of liver tissues from mice fed a LFD, HFD, or HFD supplemented with 0.1% PGA (HPGA) for 12 weeks (*n* = 6). The 46 lipid species significantly down-regulated by PGA (HPGA vs. HFD, *p* < 0.05) are categorized based on their response to the HFD (HFD vs. LFD): (**A**) 15 lipid species that were significantly up-regulated by HFD and subsequently down-regulated by PGA; (**B**) 28 lipid species that were not significantly altered by HFD but were significantly down-regulated by PGA; (**C**) 3 lipid species that were significantly down-regulated by HFD and were further suppressed by PGA. Lipid species within each panel are sorted in ascending order of their fold_change (HPGA vs. HFD). CE, cholesterol ester; Cer, ceramide; DG, diacylglycerol; HFD, high-fat diet; HPGA, HFD containing 0.1% of phloroglucinaldehyde; LPS, lysophosphatidyl serine; PE, phosphatidylethanolamine; TG, triglycerides.

In contrast, dietary intervention with PGA (HPGA group) specifically down-regulated 46 lipid metabolites relative to the HFD group (*p* < 0.05), with no significant up-regulations observed. We further categorized these 46 PGA-down-regulated lipids based on their response to the HFD to gain deeper insight into PGA’s action (Figure 2 & Supplementary Figure S1F):

Category A (Figure 2A): 15 lipid species, predominantly triglycerides, were significantly up-regulated by HFD and subsequently normalized by PGA supplementation (*p* < 0.05 for both LFD vs. HFD and HFD vs. HPGA). These lipids included LPS(18:0/0:0), LPS(18:1/0:0), TG(18:1/18:2/20:0), TG(14:0/20:0/22:3), TG(14:0/20:3/22:1), TG(14:0/18:2/22:0), TG(16:1/20:2/22:0), TG(16:0/18:0/22:1), TG(18:1/18:3/20:0), TG(16:0/18:2/22:3), DG(20:0/22:6/0:0), TG(16:0/16:1/22:3), TG(14:0/20:2/22:1), TG(18:0/18:3/20:1), and TG(16:0/16:1/20:0).

Category B (Figure 2B): 28 lipid species, unaffected by HFD (*p* > 0.05 vs. LFD), were significantly reduced by PGA (*p* < 0.05). These lipids included TG(16:1/18:1/20:0), TG(16:0/18:2/22:0), Cer(m18:1/24:0), TG(16:0/16:1/22:0), TG(14:0/20:1/22:2), TG(12:0/22:1/22:3), TG(14:0/18:0/22:1), TG(14:0/20:0/20:2), TG(16:0/18:1/22:0), TG(14:0/20:1/22:0), TG(18:0/18:0/18:1), TG(14:0/20:0/20:1), TG(18:1/20:1/20:2), TG(16:0/16:1/22:1), TG(14:0/20:1/20:1), TG(16:1/18:2/20:0), TG(14:0/20:0/22:1), TG(14:0/22:1/22:2), TG(14:0/20:3/22:2), TG(14:0/22:1/22:1), Cer(m18:1/24:1), TG(18:1/18:1/20:0), TG(18:1/20:1/20:1), TG(14:0/22:0/22:2), TG(14:0/22:1/22:3), TG(14:0/18:0/20:1), TG(14:0/20:1/20:3), and TG(14:0/20:2/20:3), sorting in ascending order of the fold_change value caused by HPGA.

Category C (Figure 2C): 3 lipid species were down-regulated by HFD (*p* < 0.05 vs. LFD) and were further suppressed by PGA (*p* < 0.05). These lipids included PE(16:1/16:1), TG(16:0/16:1/20:1), and CE(16:1), sorting in ascending order of the fold_change value caused by HPGA.

For a complete overview, the fold changes for all 46 lipid species in both the LFD vs. HFD and HFD vs. HPGA comparisons are detailed in Table 1. Lipidomic profiling revealed that HFD feeding induced a distinct remodeling of hepatic triglycerides (TGs), which was effectively reversed by HPGA supplementation. The down-regulated lipids were markedly enriched in very-long-chain fatty acids (C ≥ 22). A key finding was the specific enrichment of TGs with n-6 polyunsaturated fatty acids (PUFAs), particularly linoleic acid (18:2), at the critical sn-2 position (e.g., TG(18:1/18:2/20:0)). Concurrently, TGs containing multiple PUFAs (e.g., TG(16:0/18:2/22:3)) and those dominated by saturated and monounsaturated fatty acids showed more variable changes. KEGG pathway analysis further indicated that these differentially regulated lipids are collectively involved in critical metabolic processes, including insulin resistance, fat digestion and absorption, cholesterol metabolism, and biosynthesis of unsaturated fatty acids (Supplementary Figure S1G), underscoring the broad impact of HPGA on lipid-mediated metabolic pathways.

### 3.4. Dietary Supplement of PGA Enhanced Antioxidant and Inhibited Pro-Inflammatory Factors

**Figure 3:**
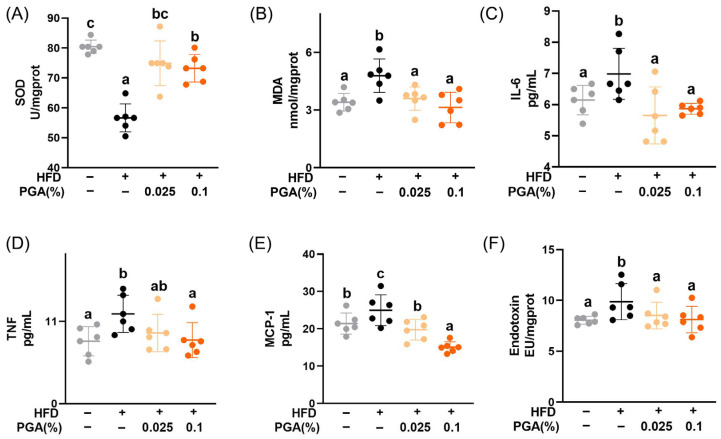
Effects of dietary supplement of PGA on antioxidant and pro-inflammatory factors. SOD activity (**A**) and MDA level (**B**) in the liver. Serum levels of IL-6 (**C**), TNF (**D**), and MCP-1 (**E**). (**F**) The level of endotoxin in the liver. Data represent mean ± SD (*n* = 6), and bars with different letters differ significantly (*p* < 0.05).

As illustrated in Figure 3, HFD consumption compromised the hepatic redox balance, which was effectively restored by PGA supplementation. Dietary PGA significantly enhanced the hepatic antioxidant capacity by increasing SOD activity (*p* < 0.05) and reducing MDA production (*p* < 0.05), a product indicative of lipid peroxidation. Concurrently, the dietary intervention with PGA markedly suppressed the HFD-induced systemic and hepatic inflammation, as evidenced by decreased levels of pro-inflammatory mediators, such as IL-6, TNF, MCP-1 and endotoxin (*p* < 0.05). However, there were limited effects on levels of IL-10, IFN-γ and IL-12p70 (*p* > 0.05, Supplementary Figure S2A–C).

### 3.5. Fifteen Types of PGA Down-Regulated Lipids Exhibit Positive Correlations with Pro-Inflammatory Markers but Negative Correlations with Antioxidant Parameters

**Figure 4:**
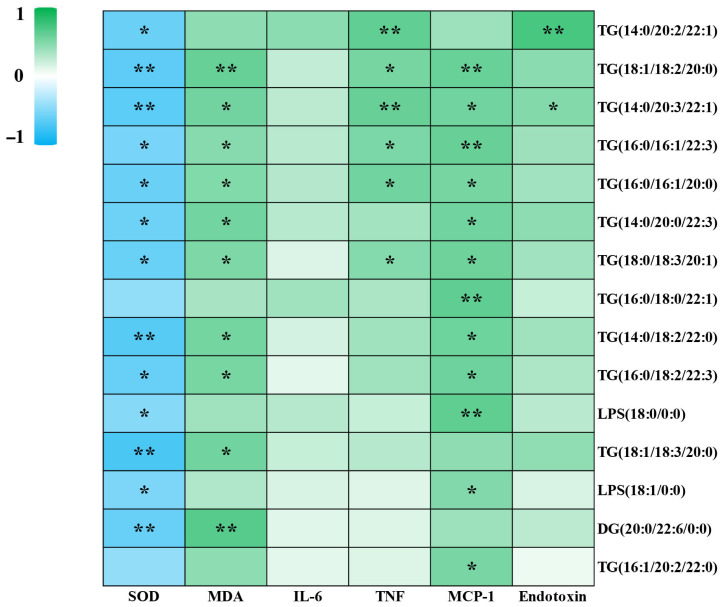
Correlation analysis between 15 types of PGA down-regulated lipids and antioxidants as well as pro-inflammatory indicators. The color intensity reflects the strength of the correlation (green, positive correlation; blue, negative correlation). Significant correlations are indicated by * *p* < 0.05 and ** *p* < 0.01.

Further correlation analysis of 15 lipid species (Section 3.3, category A), up-regulated by HFD and subsequently normalized by PGA supplementation, with the oxidative stress/inflammatory indicators described in Section 3.4 was performed. The results demonstrated that SOD levels were significantly negatively correlated (*p* < 0.05) with 13 distinct hepatic lipid species, including TG(14:0/20:2/22:1), TG(18:1/18:2/20:0), TG(14:0/20:3/22:1), TG(16:0/16:1/22:3), TG(16:0/16:1/20:0), TG(14:0/20:0/22:3), TG(18:0/18:3/20:1), TG(14:0/18:2/22:0), TG(16:0/18:2/22:3), LPS(18:0/0:0), TG(18:1/18:3/20:0), LPS(18:1/0:0) and DG(20:0/22:6/0:0). MDA levels were significantly positively correlated (*p* < 0.05) with 10 distinct hepatic lipid species, including TG(18:1/18:2/20:0), TG(14:0/20:3/22:1), TG(16:0/16:1/22:3), TG(16:0/16:1/20:0), TG(14:0/20:0/22:3), TG(18:0/18:3/20:1), TG(14:0/18:2/22:0), TG(16:0/18:2/22:3), TG(18:1/18:3/20:0) and DG(20:0/22:6/0:0). TNF levels were significantly positively correlated (*p* < 0.05) with 6 distinct hepatic lipid species, including TG(14:0/20:2/22:1), TG(18:1/18:2/20:0), TG(14:0/20:3/22:1), TG(16:0/16:1/22:3), TG(16:0/16:1/20:0) and TG(18:0/18:3/20:1). MCP-1 levels were significantly positively correlated (*p* < 0.05) with 12 distinct hepatic lipid species, including TG(18:1/18:2/20:0), TG(14:0/20:3/22:1), TG(16:0/16:1/22:3), TG(16:0/16:1/20:0), TG(14:0/20:0/22:3), TG(18:0/18:3/20:1), TG(16:0/18:0/22:1), TG(14:0/18:2/22:0), TG(16:0/18:2/22:3), LPS(18:0/0:0), LPS(18:1/0:0) and TG(16:1/20:2/22:0). Endotoxin levels were significantly positively correlated (*p* < 0.05) with 2 distinct hepatic lipid species, including TG(18:1/18:2/20:0) and TG(16:0/16:1/22:3) (Figure 4).

## 4. Discussions

The global consumption of C3G-rich berries, such as blue honeysuckle (*Lonicera caerulea* L.), has been associated with a spectrum of health benefits, particularly in mitigating metabolic syndromes [31]. A significant pharmacological challenge, however, lies in the inherently low systemic bioavailability of parent anthocyanins [22]. While strategies like enzymatic acylation [32] and advanced packaging technologies [33,34,35] aim to enhance their stability and absorption, a paradigm shift is underway, focusing on the bioactive metabolites that are ultimately responsible for the systemic effects observed after anthocyanin ingestion. According to a human pharmacokinetic study of C3G [36], protocatechuic acid (PCA), PGA, vanillic acid (VA), ferulic acid (FA) and their derivates represent the main metabolites of C3G [37]. Despite the fact that hippuric acid is also one of the major metabolite, we paid little attention to it since it is considered as an end-product with limited biological activities [38]. The beneficial effects of PCA on lipid metabolism have been well documented [29,39], whereas studies specifically investigating the role of PGA in lipid metabolism remain scarce.

Our initial in vitro screening in LO2 hepatocytes established a safe and effective concentration window for PGA, demonstrating its efficacy in reducing lipid accumulation at doses of 25–100 μM. The safe and effective concentration range of PGA (25–100 µM) identified in vitro is consistent with the active concentrations reported for other bioactive compounds [40]. Although direct extrapolation to circulating levels is complex, the significant therapeutic effects observed in vivo with dietary PGA supplementation confirm that a biologically active dose is delivered to the liver, validating the physiological relevance of our in vitro findings. It is noteworthy that a higher concentration (500 μM) inhibited cell growth, indicating a defined cytotoxic threshold. This finding underscores the importance of dosage considerations for any potential application.

Our study provided compelling evidence that PGA, a key metabolite derived from common dietary anthocyanins, conferred robust protection against HFD-induced MAFLD. The initial hallmark of MAFLD is often hepatic steatosis, frequently associated with overweight, though non-obese phenotypes are increasingly recognized [41,42,43]. In our model, PGA supplementation effectively counteracted HFD-induced body weight gain and intraperitoneal adiposity. More importantly, it ameliorated key hallmarks of MAFLD, including hepatic lipid accumulation, elevated serum levels of liver transaminases (AST and ALT), and systemic insulin resistance [44]. The result of different daily energy intake was crucial for interpreting our results and underscored the direct metabolic role of PGA. First, it demonstrated that the HFD-induced MAFLD phenotype at present study was primarily driven by diet composition (high fat) rather than hyperphagia (excessive eating). It seemed that the high fat content in HFD, LPGA and HPGA group might reduce the overall palatability, leading to a decrease in gram-weight food intake that resulted in energy intake similar to that of low-fat diet controls. Importantly, the fact that the HPGA group showed profound alleviation of MAFLD without any reduction in energy intake compared to the HFD group provided strong evidence that the therapeutic effects of the high-dose PGA were independent of caloric intake and were mediated by direct metabolic mechanisms. The case of the LPGA group was particularly compelling. Despite consuming significantly more calories than the HFD group, the LPGA mice still exhibited clear metabolic improvements. This indicated that even the low dose of PGA was potent enough to counteract the detrimental effects of both a high-fat diet and an additional caloric load, powerfully underscoring its robust bioactivity. The profound reduction in intrahepatic lipid content, a cornerstone of MAFLD pathology [42], prompted us to delve deeper into the lipidomic landscape. Thus, the LO2 cell model served as a predictive screening tool, the results of which were directly translated and mechanistically explained in the whole-animal model, underscoring the robustness and translational relevance of our findings. 

Our untargeted lipidomics analysis offered a molecular rationale for these observations, revealing a significant down-regulation of 46 hepatic lipid species upon PGA intervention, with triglycerides (TGs) being the predominant class affected. These 46 PGA-down-regulated lipids were categorized as three sections: 15 lipid species as rescued lipids represent lipids directly involved in HFD-induced pathology that are effectively countered by PGA; 28 lipid species as newly modulated lipids suggests that PGA’s lipid-lowering effect extends beyond simply reversing HFD-induced changes, potentially modulating basal lipid metabolism; and the remaining 3 lipid species as co-suppressed lipids indicate a synergistic or additive effect by PGA. PGA remodeled the hepatic lipidome by reducing triglycerides enriched with very-long-chain and saturated fatty acids [45], particularly those with pro-lipotoxic SFA-PUFA motifs, thereby alleviating the key drivers of oxidative stress and inflammation. KEGG pathway analysis further positioned these structural-specific lipid reductions within a coordinated amelioration of insulin signaling, fat absorption, and cholesterol metabolism, underscoring the multi-faceted hepatic protection conferred by PGA. This finding was particularly relevant as hepatic TG accumulation was not merely a static storage issue but was a recognized key driver of lipotoxicity and disease progression [46]. The specific reduction in TGs suggested that PGA might regulate key pathways involved in hepatic lipid metabolism. Future studies employing transcriptional profiling could investigate the associated changes in gene expression to elucidate the precise molecular mechanisms underlying these lipid-lowering effects.

Beyond lipid modulation, our findings illuminated two critical mechanistic pathways through which PGA exerted its protective effects: enhanced antioxidant defense and suppression of chronic inflammation. The observed increase in hepatic SOD activity and concurrent decrease in MDA levels indicated a bolstering of the endogenous antioxidant system and a reduction in lipid peroxidation, respectively. Oxidative stress is a well-established driver of MAFLD [47], promoting hepatocyte injury and fibrosis. It was plausible that PGA, by virtue of its phloroglucinol structure, might directly scavenge reactive oxygen species (ROS) or, as a testable hypothesis, upregulate the Nrf2 antioxidant signaling pathway, a common mechanism for many polyphenols [48]. While the classic combination of SOD and MDA used in this study confirmed the antioxidant effect of PGA, future research could elucidate its detailed molecular targets by examining key enzymes such as catalase and components of the glutathione system. Chronic inflammation is also a critical event in the progression of MAFLD [49]. Concurrently, PGA demonstrated a potent anti-inflammatory effect, significantly reducing serum levels of key pro-inflammatory cytokines (IL-6, TNF, MCP-1 [50]) and hepatic endotoxin, the primary component of which consists of lipopolysaccharides known for their pro-inflammatory and MAFLD-promoting properties [51]. This anti-inflammatory effect was supported by the observed reduction in hepatic triglycerides containing n-6 polyunsaturated fatty acids at the sn-2 position (Table 1), which serve as key precursors in the biosynthesis of pro-inflammatory lipid mediators [52,53]. The reduction in these critical mediators suggests that PGA may interfere with key inflammatory signaling cascades, potentially including the NF-κB pathway, a notion that warrants further investigation.

The most integrative insight from our study comes from the correlation analysis, which weaved these individual threads into a coherent mechanistic narrative. The significant positive correlations between specific PGA-down-regulated lipids (e.g., TG(18:1/18:2/20:0), TG(14:0/20:3/22:1)) and markers of oxidative stress (MDA) and inflammation (TNF, MCP-1) suggested a vicious cycle wherein these lipids were not merely inert deposits but active contributors to a pro-oxidant and pro-inflammatory hepatic milieu. Conversely, their negative correlation with the antioxidant SOD implied that their accumulation overwhelmed the cellular defense systems. Therefore, we hypothesize that PGA may contribute to reducing specific harmful lipid species. By alleviating lipotoxicity, PGA may concurrently mitigate the associated oxidative stress and inflammation, which in turn could foster a more favorable metabolic environment that is consistent with the observed reduction in aberrant lipid accumulation [54,55]. This break in the “lipotoxicity-oxidative stress-inflammation” axis is consistent with a fundamental mechanism that may underpin PGA’s efficacy.

It should be noted that this study was conducted exclusively in male mice. Given the known sexual dimorphism in lipid metabolism and MAFLD progression, future studies in female models are warranted. Furthermore, while this study focused on the early stages of MAFLD, future studies employing longer-term or more aggressive dietary models are warranted to determine the efficacy of PGA in preventing or reversing hepatic fibrosis, a key milestone in disease progression. While this study provides robust evidence for the efficacy of PGA, we note that it did not include a positive control group treated with a current standard-of-care agent for MAFLD/NAFLD. Future comparative efficacy studies will be important to fully contextualize the therapeutic potential of PGA relative to existing pharmacological approaches.

Overall, our findings indicate that the health benefits of a diet rich in anthocyanin-containing foods were not merely due to the parent compounds but are also mediated by metabolites like PGA. This understanding is crucial for developing more effective dietary strategies and functional foods. Given its smaller size and stability compared to C3G, PGA itself could be considered a promising candidate for nutraceutical applications or as a biomarker for the efficacy of anthocyanin-rich diets. Future translation of these findings requires determining the dietary anthocyanin intake needed to achieve bioactive PGA levels in humans and evaluating its safety and potential for synergistic use with existing therapies. The results in this study suggest that the protective effects of dietary anthocyanins against MAFLD could be mediated, at least in part, by the bioactivity of PGA. Supplementation with this bioactive metabolite alleviated hepatic steatosis, oxidative stress, and inflammation, underscoring its potential role in the future of interventions for metabolic liver diseases.

## 5. Conclusions

In conclusion, dietary supplementation with phloroglucinaldehyde (PGA), a key metabolite derived from polyphenol cyanidin-3-glucoside (C3G), prevented MAFLD via the enhancement of antioxidant and anti-inflammatory mechanisms.

## Figures and Tables

**Table 1:** Comprehensive list of the 46 hepatic lipid species down-regulated by PGA supplementation and their fold changes in relevant comparisons.

Compounds	LM ID ^①^	Chain Length ^②^	Structural Motifs ^③^	PUFA Omega Series ^④^	Fold_Change ^⑤^	
					**LFD vs. HFD**	**HFD vs. HPGA**
LPS (18:0/0:0)	LMGP03050006	Long chain	SFA	N/A	3.93	0.25
LPS (18:1/0:0)	LMGP03050001	Long chain	MUFA	N/A	4.50	0.33
TG (18:1/18:2/20:0)	LMGL03010478	Long chain	MUFA/PUFA/SFA	n-6 (sn-2)	2.82	0.37
TG (14:0/20:0/22:3)	LMGL03014490	Very long chain	SFA/SFA/PUFA	n-6 (sn-3)	3.22	0.37
TG (14:0/20:3/22:1)	LMGL03014521	Very long chain	SFA/PUFA/MUFA	n-6 (sn-2)	3.20	0.40
TG (14:0/18:2/22:0)	LMGL03014394	Very long chain	SFA/PUFA/SFA	n-6 (sn-2)	3.96	0.42
TG (16:1/20:2/22:0)	LMGL03010964	Very long chain	MUFA/PUFA/SFA	n-6 (sn-2)	2.45	0.44
TG (16:0/18:0/22:1)	LMGL03010515	Very long chain	SFA/SFA/MUFA	N/A	2.45	0.45
TG (18:1/18:3/20:0)	LMGL03010532	Long chain	MUFA/PUFA/SFA	n-3 (sn-2)	5.38	0.45
TG (16:0/18:2/22:3)	LMGL03010700	Very long chain	SFA/PUFA/PUFA	n-6 (sn-2 & sn-3)	3.08	0.46
DG (20:0/22:6/0:0)	LMGL02010257	Very long chain	SFA/PUFA	n-3 (sn-2)	2.45	0.48
TG (16:0/16:1/22:3)	LMGL03010369	Very long chain	SFA/MUFA/PUFA	n-6 (sn-3)	2.22	0.48
TG (14:0/20:2/22:1)	LMGL03014511	Very long chain	SFA/PUFA/MUFA	n-6 (sn-2)	2.75	0.49
TG (18:0/18:3/20:1)	LMGL03010537	Long chain	SFA/PUFA/MUFA	n-3 (sn-2)	2.50	0.49
TG (16:0/16:1/20:0)	LMGL03010095	Long chain	SFA/MUFA/SFA	N/A	2.40	0.50
TG (16:1/18:1/20:0)	LMGL03010231	Long chain	MUFA/MUFA/SFA	N/A	1.02	0.19
TG (16:0/18:2/22:0)	LMGL03010568	Very long chain	SFA/PUFA/SFA	n-6 (sn-2)	0.88	0.24
Cer (m18:1/24:0)	LMSP00000008	Very long chain	SFA/MUFA	N/A	1.95	0.27
TG (16:0/16:1/22:0)	LMGL03010285	Very long chain	SFA/MUFA/SFA	N/A	1.43	0.27
TG (14:0/20:1/22:2)	LMGL03014501	Very long chain	SFA/MUFA/PUFA	n-6 (sn-3)	1.48	0.27
TG (12:0/22:1/22:3)	LMGL03013658	Very long chain	SFA/MUFA/PUFA	n-6 (sn-3)	1.36	0.27
TG (14:0/18:0/22:1)	LMGL03014356	Very long chain	SFA/SFA/MUFA	N/A	0.81	0.28
TG (14:0/20:0/20:2)	LMGL03014482	Long chain	SFA/SFA/PUFA	n-6 (sn-3)	2.72	0.30
TG (16:0/18:1/22:0)	LMGL03010511	Very long chain	SFA/MUFA/SFA	N/A	1.41	0.31
TG (14:0/20:1/22:0)	LMGL03014499	Very long chain	SFA/MUFA/SFA	N/A	0.79	0.31
TG (18:0/18:0/18:1)	LMGL03010188	Long chain	SFA/SFA/MUFA	N/A	1.87	0.34
TG (14:0/20:0/20:1)	LMGL03014481	Long chain	SFA/SFA/MUFA	N/A	1.25	0.36
TG (18:1/20:1/20:2)	LMGL03010861	Long chain	MUFA/MUFA/PUFA	n-6 (sn-3)	1.50	0.36
TG (16:0/16:1/22:1)	LMGL03010325	Very long chain	SFA/MUFA/MUFA	N/A	0.98	0.37
TG (14:0/20:1/20:1)	LMGL03012800	Long chain	SFA/MUFA/MUFA	N/A	1.21	0.37
TG (16:1/18:2/20:0)	LMGL03010263	Long chain	MUFA/PUFA/SFA	n-6 (sn-2)	0.65	0.37
TG (14:0/20:0/22:1)	LMGL03014488	Very long chain	SFA/SFA/MUFA	N/A	2.10	0.37
TG (14:0/22:1/22:2)	LMGL03014557	Very long chain	SFA/MUFA/PUFA	n-6 (sn-3)	0.80	0.37
TG (14:0/20:3/22:2)	LMGL03014522	Very long chain	SFA/PUFA/PUFA	n-6 (sn-2 & sn-3)	1.49	0.38
TG (14:0/22:1/22:1)	LMGL03012807	Very long chain	SFA/MUFA/MUFA	N/A	1.07	0.38
Cer (m18:1/24:1)	LMSP00000010	Very long chain	MUFA/MUFA	N/A	0.99	0.40
TG (18:1/18:1/20:0)	LMGL03010428	Long chain	MUFA/MUFA/SFA	N/A	1.57	0.40
TG (18:1/20:1/20:1)	LMGL03010787	Long chain	MUFA/MUFA/MUFA	N/A	0.57	0.42
TG (14:0/22:0/22:2)	LMGL03014552	Very long chain	SFA/SFA/PUFA	n-6 (sn-3)	1.09	0.42
TG (14:0/22:1/22:3)	LMGL03014558	Very long chain	SFA/MUFA/PUFA	n-6 (sn-3)	1.78	0.44
TG (14:0/18:0/20:1)	LMGL03014349	Long chain	SFA/SFA/MUFA	N/A	0.98	0.45
TG (14:0/20:1/20:3)	LMGL03014495	Long chain	SFA/MUFA/PUFA	n-6 (sn-3)	1.24	0.48
TG (14:0/20:2/20:3)	LMGL03014506	Long chain	SFA/PUFA/PUFA	n-6 (sn-2 & sn-3)	1.68	0.49
PE (16:1/16:1)	LMGP02010108	Long chain	MUFA/MUFA	N/A	0.11	0.40
TG (16:0/16:1/20:1)	LMGL03010114	Long chain	SFA/MUFA/MUFA	N/A	0.45	0.40
CE (16:1)	LMST01020006	Long chain	MUFA	N/A	0.29	0.48

Notes. ^①^ Details of the compounds are available at http://www.lipidmaps.org (accessed on 14 July 2025). ^②^ Chain length was classified by carbon number: C14–20 as long-chain (black), C ≥ 22 as very long-chain (red). ^③^ Structural motifs were determined by fatty acid saturation, double-bond count, and sn-positions (sn indicates glycerol backbone positions); saturated fatty acids (red), polyunsaturated fatty acids (blue), monounsaturated fatty acids (black). ^④^ Omega series was assigned based on the methyl-terminal double-bond position of PUFA, with sn-position indicated; n-6 (red), n-3 (blue). ^⑤^ Fold-changes of differential lipid metabolites are shown for each comparison group (*n* = 6): significant up-regulation (red), significant down-regulation (blue), non-significant changes (black). CE, cholesterol ester; Cer, ceramide; DG, diacylglycerol; HFD, high-fat diet; HPGA, HFD containing 0.1% of phloroglucinaldehyde; LFD, low-fat diet; LM, lipid maps; LPS, lysophosphatidyl serine; MUFA, monounsaturated fatty acids; N/A, not applicable; PE, phosphatidylethanolamine; PUFA, polyunsaturated fatty acids; SFA, saturated fatty acids; TG, triglycerides.

## Data Availability

The original contributions presented in the study are included in the article/Supplementary Materials, further inquiries can be directed to the corresponding author.

## Supplementary Materials

The following supporting information can be downloaded at https://www.mdpi.com/article/10.3390/foods15030437/s1, Figure S1: Energy intake, adipocyte counts, serum biochemistry, and lipidomic profiling in experimental groups; Figure S2: Serum cytokine profile following PGA dietary supplementation (IL-10, IFN-γ, and IL-12p70); Table S1: Diet composition of LFD and HFD; Table S2: Hepatic lipid metabolites modulated by HFD.

## References

[1] Amini-Salehi E., Letafatkar N., Norouzi N., Joukar F., Habibi A., Javid M., Sattari N., Khorasani M., Farahmand A., Tavakoli S. (2024). Global Prevalence of Nonalcoholic Fatty Liver Disease: An Updated Review Meta-Analysis comprising a Population of 78 million from 38 Countries. Arch. Med. Res..

[2] Setayesh T., Hu Y., Vaziri F., Wei D., Wan Y.-J.Y. (2024). The spatial impact of a Western diet in enriching Galectin-1-regulated Rho, ECM, and SASP signaling in a novel MASH-HCC mouse model. Biomark. Res..

[3] Buzzetti E., Pinzani M., Tsochatzis E.A. (2016). The multiple-hit pathogenesis of non-alcoholic fatty liver disease (NAFLD). Metabolism.

[4] Sakurai Y., Kubota N., Yamauchi T., Kadowaki T. (2021). Role of Insulin Resistance in MAFLD. Int. J. Mol. Sci..

[5] Yang M., Liu X., Li Q., Liu J., Wang B. (2025). Insulin resistance as potential mediator linking ApoB/ApoA1 to MAFLD, but not inflammation. Ther. Adv. Endocrinol. Metab..

[6] Truong X.T., Lee D.H. (2025). Hepatic Insulin Resistance and Steatosis in Metabolic Dysfunction-Associated Steatotic Liver Disease: New Insights into Mechanisms and Clinical Implications. Diabetes Metab. J..

[7] Geng Y., Faber K.N., de Meijer V.E., Blokzijl H., Moshage H. (2021). How does hepatic lipid accumulation lead to lipotoxicity in non-alcoholic fatty liver disease?. Hepatol. Int..

[8] Zhang Z., Yang H., Han F., Guo P. (2025). Reactive Oxygen Species as Key Molecules in the Pathogenesis of Alcoholic Fatty Liver Disease and Nonalcoholic Fatty Liver Disease: Future Perspectives. Curr. Issues Mol. Biol..

[9] Peiseler M., Schwabe R., Hampe J., Kubes P., Heikenwälder M., Tacke F. (2022). Immune mechanisms linking metabolic injury to inflammation and fibrosis in fatty liver disease—Novel insights into cellular communication circuits. J. Hepatol..

[10] Tan E., Danpanichkul P., Yong J., Yu Z., Tan D., Lim W., Koh B., Lim R., Tham E., Mitra K. (2024). Liver Cancer in 2021: Global Burden of Disease Study. J. Hepatol..

[11] Liu Y., Wang J., Jin R., Xu Z., Zhao X., Li Y., Zhao Y., Wu Z., Guo X., Tao L. (2024). Associations of Metabolic Dysfunction-Associated Fatty Liver Disease With Peripheral Artery Disease: Prospective Analysis in the UK Biobank and ARIC Study. J. Am. Heart Assoc..

[12] Pennisi G., Infantino G., Celsa C., Di Maria G., Enea M., Vaccaro M., Cannella R., Ciccioli C., La Mantia C., Mantovani A. (2024). Clinical outcomes of MAFLD versus NAFLD: A meta-analysis of observational studies. Liver Int..

[13] Lago-Sampedro A., Oualla-Bachiri W., García-Serrano S., Maldonado-Araque C., Valdés S., Doulatram-Gamgaram V., Olveira G., Delgado E., Chaves F.J., Castaño L. (2024). Protective Effect of High Adherence to Mediterranean Diet on the Risk of Incident Type-2 Diabetes in Subjects with MAFLD: The Di@bet.es Study. Nutrients.

[14] Stachowicz A., Czepiel K., Wiśniewska A., Stachyra K., Ulatowska-Białas M., Kuśnierz-Cabala B., Surmiak M., Majka G., Kuś K., Wood M.E. (2024). Mitochondria-targeted hydrogen sulfide donor reduces fatty liver and obesity in mice fed a high fat diet by inhibiting de novo lipogenesis and inflammation via mTOR/SREBP-1 and NF-κB signaling pathways. Pharmacol. Res..

[15] Zakaria Z., Othman Z.A., Suleiman J.B., Jalil N.A.C., Ghazali W.S.W., Mohamed M. (2021). Protective and Therapeutic Effects of Orlistat on Metabolic Syndrome and Oxidative Stress in High-Fat Diet-Induced Metabolic Dysfunction-Associated Fatty Liver Disease (MAFLD) in Rats: Role on Nrf2 Activation. Vet. Sci..

[16] Xiong Y., Huang X., Li Y., Nie Y., Yu H., Shi Y., Xue J., Ji Z., Rong K., Zhang X. (2024). Integrating larval zebrafish model and network pharmacology for screening and identification of edible herbs with therapeutic potential for MAFLD: A promising drug Smilax glabra Roxb. Food Chem..

[17] Wang R., Yan R., Jiao J., Li F., Zhang H., Chang Z., Wei H., Yan S., Li J. (2024). Fruit and vegetable intake and the risk of non-alcoholic fatty liver disease: A meta-analysis of observational studies. Front. Nutr..

[18] Wang Q., Zhang Y., Lu R., Zhao Q., Gao Y. (2024). The multiple mechanisms and therapeutic significance of rutin in metabolic dysfunction-associated fatty liver disease (MAFLD). Fitoterapia.

[19] Yang M., Xia L., Song J., Hu H., Zang N., Yang J., Zou Y., Wang L., Zheng X., He Q. (2023). Puerarin ameliorates metabolic dysfunction-associated fatty liver disease by inhibiting ferroptosis and inflammation. Lipids Health Dis..

[20] Zhao Y., Jiang C., Lu J., Sun Y., Cui Y. (2023). Research progress of proanthocyanidins and anthocyanidins. Phytother. Res..

[21] Zhao R., Xiang B., Dolinsky V.W., Xia M., Shen G.X. (2021). Saskatoon berry powder reduces hepatic steatosis and insulin resistance in high fat-high sucrose diet-induced obese mice. J. Nutr. Biochem..

[22] Deepa P., Hong M., Sowndhararajan K., Kim S. (2023). A Review of the Role of an Anthocyanin, Cyanidin-3-O-β-glucoside in Obesity-Related Complications. Plants.

[23] Wang L., Lan W., Chen D. (2024). Blueberry (*Vaccinium* spp.) Anthocyanins and Their Functions, Stability, Bioavailability, and Applications. Foods.

[24] Redha A.A., Kodikara C., Cozzolino D. (2024). Does Encapsulation Improve the Bioavailability of Polyphenols in Humans? A Concise Review Based on In Vivo Human Studies. Nutrients.

[25] Płatosz N., Bączek N., Topolska J., Szawara-Nowak D., Skipor J., Milewski S., Wiczkowski W. (2020). Chokeberry anthocyanins and their metabolites ability to cross the blood-cerebrospinal fluid barrier. Food Chem..

[26] Wang B., Tang X., Mao B., Zhang Q., Tian F., Zhao J., Cui S., Chen W. (2022). Anti-aging effects and mechanisms of anthocyanins and their intestinal microflora metabolites. Crit. Rev. Food Sci. Nutr..

[27] Mostafa H., Behrendt I., Meroño T., González-Domínguez R., Fasshauer M., Rudloff S., Andres-Lacueva C., Kuntz S. (2022). Plasma anthocyanins and their metabolites reduce in vitro migration of pancreatic cancer cells, PANC-1, in a FAK- and NF-kB dependent manner: Results from the ATTACH-study a randomized, controlled, crossover trial in healthy subjects. Biomed. Pharmacother..

[28] Wang B., Tang X., Mao B., Zhang Q., Tian F., Zhao J., Chen W., Cui S. (2024). Comparison of the hepatoprotection of intragastric and intravenous cyanidin-3-glucoside administration: Focus on the key metabolites and gut microbiota modulation. Food Funct..

[29] Tan J., Hu R., Gong J., Fang C., Li Y., Liu M., He Z., Hou D.-X., Zhang H., He J. (2023). Protection against Metabolic Associated Fatty Liver Disease by Protocatechuic Acid. Gut Microbes.

[30] Matthews D.R., Hosker J.P., Rudenski A.S., Naylor B.A., Treacher D.F., Turner R.C. (1985). Homeostasis model assessment: Insulin resistance and beta-cell function from fasting plasma glucose and insulin concentrations in man. Diabetologia.

[31] Liu M., Tan J., He Z., He X., Hou D.-X., He J., Wu S. (2018). Inhibitory effect of blue honeysuckle extract on high-fat-diet-induced fatty liver in mice. Anim. Nutr..

[32] Li Z., Teng W., Xie X., Bao Y., Xu A., Sun Y., Yang B., Tian J., Li B. (2024). Enzymatic acylation of cyanidin-3-O-glucoside with aromatic and aliphatic acid methyl ester: Structure-stability relationships of acylated derivatives. Food Res. Int..

[33] Zhong H., Hussain M., Hussain K., Wang L., Abdullah, Qayum A., Hamed Y.S., Guan R. (2024). Nanoliposomes a future based delivery vehicle of cyanidin-3-O-glucoside against major chronic disease. Crit. Rev. Food Sci. Nutr..

[34] Song W., Yuan Q., Xie Y., Wang Y., Deng D., Guo H. (2024). Formulation and characterization of nanocapsules loaded with roselle anthocyanins extract and enhancement of anthocyanins bioaccessibility. Food Chem..

[35] Jiang Q., Sun Y., Si X., Cui H., Li J., Bao Y., Wang L., Li B. (2024). Anthocyanin-loaded milk-derived extracellular vesicles nano-delivery system: Stability, mucus layer penetration, and pro-oxidant effect on HepG2 cells. Food Chem..

[36] De Ferrars R.M., Czank C., Zhang Q., Botting N.P., Kroon P., Cassidy A., Kay C. (2014). The pharmacokinetics of anthocyanins and their metabolites in humans. Br. J. Pharmacol..

[37] Tan J., Li Y., Hou D.-X., Wu S. (2019). The Effects and Mechanisms of Cyanidin-3-Glucoside and Its Phenolic Metabolites in Maintaining Intestinal Integrity. Antioxidants.

[38] De Simone G., Balducci C., Forloni G., Pastorelli R., Brunelli L. (2021). Hippuric acid: Could became a barometer for frailty and geriatric syndromes?. Ageing Res. Rev..

[39] Ding H., Liu J., Chen Z., Huang S., Yan C., Kwek E., He Z., Zhu H., Chen Z.-Y. (2024). Protocatechuic acid alleviates TMAO-aggravated atherosclerosis via mitigating inflammation, regulating lipid metabolism, and reshaping gut microbiota. Food Funct..

[40] He Z., Uto T., Tanigawa S., Sakao K., Kumamoto T., Xie K., Pan X., Wu S., Yang Y., Komatsu M. (2024). Fisetin is a selective adenosine triphosphate-competitive inhibitor for mitogen-activated protein kinase kinase 4 to inhibit lipopolysaccharide-stimulated inflammation. Biofactors.

[41] Eslam M., El-Serag H.B., Francque S., Sarin S.K., Wei L., Bugianesi E., George J. (2022). Metabolic (dysfunction)-associated fatty liver disease in individuals of normal weight. Nat. Rev. Gastroenterol. Hepatol..

[42] Cheng Y.-M., Wang S.-W., Wang C.-C., Kao J.-H. (2024). Clinical characteristics of lean metabolic-associated fatty liver disease and the impact of concurrent diabetes mellitus. Tzu Chi Med. J..

[43] Quek J., Chan K.E., Wong Z.Y., Tan C., Tan B., Lim W.H., Tan D.J.H., Tang A.S.P., Tay P., Xiao J. (2022). Global prevalence of non-alcoholic fatty liver disease and non-alcoholic steatohepatitis in the overweight and obese population: A systematic review and meta-analysis. Lancet Gastroenterol. Hepatol..

[44] Schübel R., Nonnenmacher T., Sookthai D., Maldonado S.G., Sowah S.A., von Stackelberg O., Schlett C.L., Grafetstätter M., Nabers D., Johnson T. (2019). Similar Weight Loss Induces Greater Improvements in Insulin Sensitivity and Liver Function among Individuals with NAFLD Compared to Individuals without NAFLD. Nutrients.

[45] Zou Y., Tian L., Pei L., Hao J., Chen T., Qi J., Qiu J., Xu Y., Hu X., Chen L. (2025). SFAs facilitates ceramide’s de novo synthesis via TLR4 and intensifies hepatocyte lipotoxicity. Int. Immunopharmacol..

[46] Zhang X., Huang C., Li X., Shangguan Z., Wei W., Liu S., Yang S., Liu Y. (2020). HFD and HFD-provoked hepatic hypoxia act as reciprocal causation for NAFLD via HIF-independent signaling. BMC Gastroenterol..

[47] Tutino V., De Nunzio V., Donghia R., Caruso E.A., Cisternino A.M., Iacovazzi P.A., Mastrosimini A.M., Fernandez E.A., Giannuzzi V., Notarnicola M. (2024). Significant Increase in Oxidative Stress Indices in Erythrocyte Membranes of Obese Patients with Metabolically-Associated Fatty Liver Disease. J. Pers. Med..

[48] Wu S., Yano S., Hisanaga A., He X., He J., Sakao K., Hou D.X. (2017). Polyphenols from Lonicera caerulea L. berry attenuate experimental nonalcoholic steatohepatitis by inhibiting proinflammatory cytokines productions and lipid peroxidation. Mol. Nutr. Food Res..

[49] Hu W., Luo L., Li M., Xiong X., Huang W., Huang Y., Sun J., Ding H., Yu H. (2024). Anti-inflammatory diet reduces risk of metabolic dysfunction-associated fatty liver disease among US adults: A nationwide survey. Scand. J. Gastroenterol..

[50] Ji X., Yang L., Zhang Z., Zhang K., Chang N., Zhou X., Hou L., Yang L., Li L. (2020). Sphingosine 1-phosphate/microRNA-1249-5p/MCP-1 axis is involved in macrophage-associated inflammation in fatty liver injury in mice. Eur. J. Immunol..

[51] Schirone L., Overi D., Carpino G., Carnevale R., De Falco E., Nocella C., D’amico A., Bartimoccia S., Cammisotto V., Castellani V. (2024). Oleuropein, a Component of Extra Virgin Olive Oil, Improves Liver Steatosis and Lobular Inflammation by Lipopolysaccharides-TLR4 Axis Downregulation. Int. J. Mol. Sci..

[52] Rutting S., Papanicolaou M., Xenaki D., Wood L.G., Mullin A.M., Hansbro P.M., Oliver B.G. (2018). Dietary ω-6 polyunsaturated fatty acid arachidonic acid increases inflammation, but inhibits ECM protein expression in COPD. Respir. Res..

[53] Hayashi D., Mouchlis V.D., Dennis E.A. (2021). Omega-3 versus Omega-6 fatty acid availability is controlled by hydrophobic site geometries of phospholipase A_2_s. J. Lipid Res..

[54] Jiang L., Yi R., Chen H., Wu S. (2024). Quercetin alleviates metabolic-associated fatty liver disease by tuning hepatic lipid metabolism, oxidative stress and inflammation. Anim. Biotechnol..

[55] Farzanegi P., Dana A., Ebrahimpoor Z., Asadi M., Azarbayjani M.A. (2019). Mechanisms of beneficial effects of exercise training on non-alcoholic fatty liver disease (NAFLD): Roles of oxidative stress and inflammation. Eur. J. Sport Sci..

