# Supplementary material for: Phloroglucinaldehyde Alleviates High-Fat-Diet-Induced MAFLD via Its Antioxidant and Anti-Inflammatory Properties
Source: Foods. 2026 Jan 25;15(3):437. doi: 10.3390/foods15030437 (PMC12896450; doi:10.3390/foods15030437)
Supplement: Supplementary file 1 [file foods-15-00437-s001.zip › Supplementary Figure.pdf]

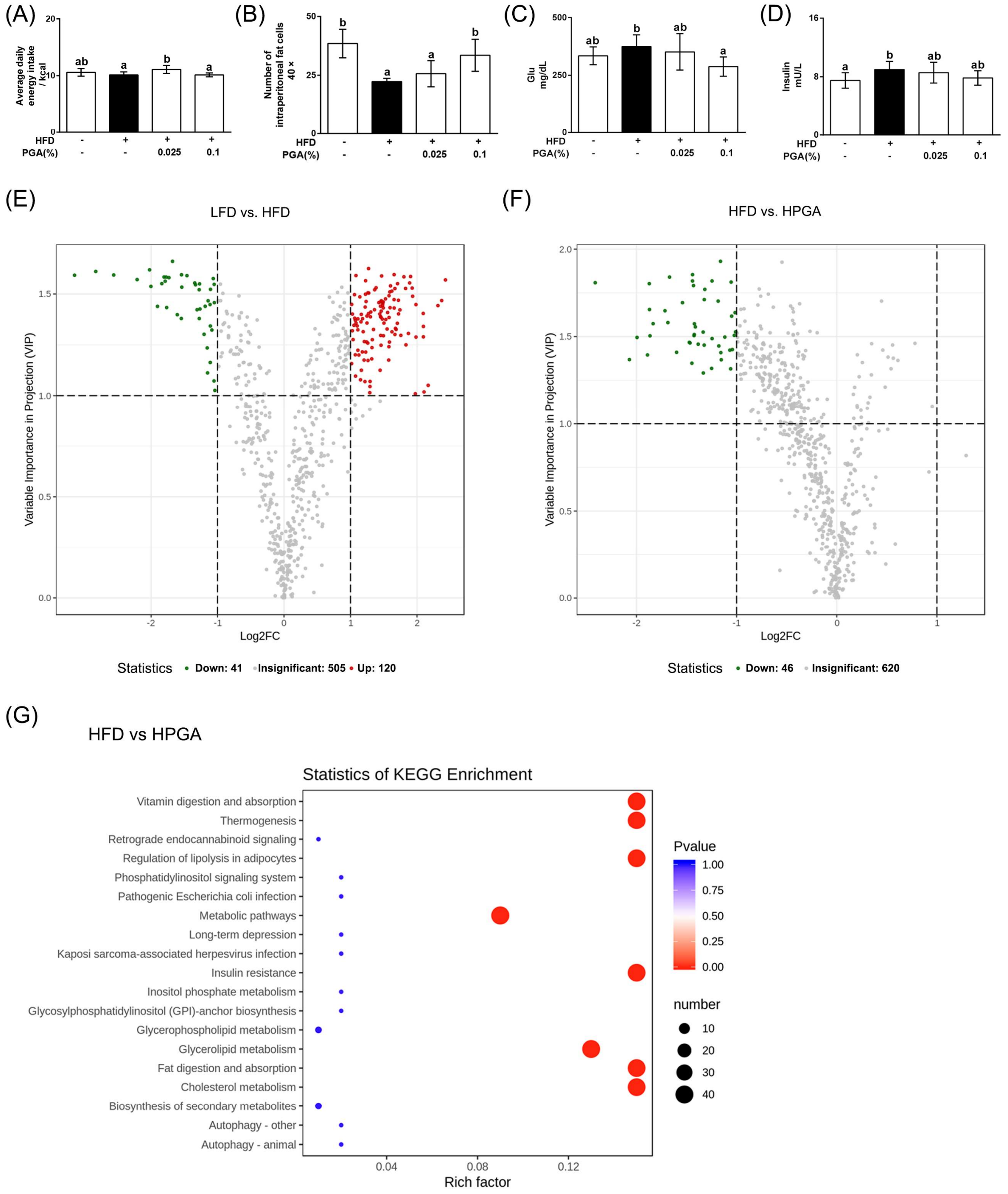

Supplementary Figure S1. (A) Average daily energy intake in mouse model. **(B)** Counts of intraperitoneal adipocytes in a fixed ×40 objective lens. Levels of glucose **(C)** and insulin **(D)** in serum. Volcanic plot exhibited different lipids metabolites between different groups, such as LFD group vs HFD group **(E)** and HFD group vs HPGA group **(F)**. **(G)** KEGG exhibited significant lipid metabolites enriched different pathways.

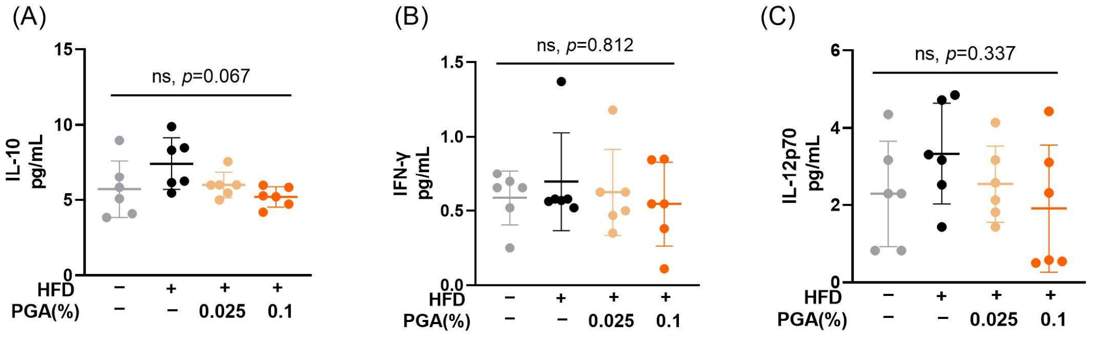

Supplementary Figure S2. Serum cytokine profile following PGA dietary supplementation (A: IL-10, B: IFN- γ , and C: IL-12p70);
